# Closure of maxillary lateral incisor agenesis space in unilateral cleft lip and palate: a digital model assessment

**DOI:** 10.1590/2177-6709.28.1.e2321331.oar

**Published:** 2023-04-14

**Authors:** Aura Sofia Caceres MANFIO, Daniela GARIB, Carlos FLORES-MIR, José Roberto Pereira LAURIS, Rodrigo TEIXEIRA, Ana Lúcia Pompéia Fraga de ALMEIDA

**Affiliations:** 1 Universidade de São Paulo, Hospital de Reabilitação de Anomalias Craniofaciais (Bauru/SP, Brazil).; 2 University of Alberta, School of Dentistry (Edmonton, AB, Canada).; 3 Universidade de São Paulo, Faculdade de Odontologia de Bauru (Bauru/SP, Brazil).

**Keywords:** Digital models, Agenesis, Cleft lip and palate, Esthetics

## Abstract

**Objective:**

To compare dental positional and gingival parameters of maxillary anterior teeth in unilateral cleft lip and palate (UCLP) after orthodontic treatment with canine substitution due to lateral incisor agenesis.

**Methods:**

This split-mouth study comprised 57 subjects with UCLP (31 male, 26 female) and agenesis of maxillary lateral incisor at the cleft side, from a single center. Canine substitution was completed after the secondary alveolar bone graft. Dental models were taken between 2 to 6 months after debonding (mean age: 20.4 years). The following variables were measured in the maxillary anterior teeth: crown height, width, proportion, and symmetry, as well as steps between incisal edges, gingival margins, tooth mesiodistal angulation and labiolingual inclination. Paired *t*-tests with Bonferroni *post-hoc* correction was used for comparisons between cleft and non-cleft sides (*p*<0.05).

**Results:**

At the cleft side, canines replacing missing lateral incisors had a higher crown height (0.77mm) and an increased width (0.67mm), and first premolars showed a shorter crown height (1.39mm). Asymmetries were observed in the gingival level of central and lateral incisors, with a greater clinical crown at the cleft side (0.61 and 0.81mm, respectively). Cleft side central incisors were more upright than their contralaterals (2.12º).

**Conclusions:**

Maxillary anterior teeth demonstrated positional, size and gingival height differences between cleft and non-cleft side after space closure of maxillary lateral incisor agenesis. Slight asymmetries in tooth position and gingival margin in the maxillary anterior teeth should be expected after orthodontic treatment in UCLP patients.

## RESUMO

**Objetivo:**

O presente trabalho teve como objetivo comparar parâmetros dentários posicionais e gengivais de dentes anteriores superiores em pacientes com fissura labiopalatina transforame unilateral (FLPTU) após tratamento ortodôntico com substituição de incisivo lateral ausente por canino, devido a agenesia.

**Métodos:**

Esse estudo de boca dividida reuniu uma amostra, de um mesmo centro especializado de tratamento, de 57 indivíduos com FLPTU (31 homens, 26 mulheres) e agenesia do incisivo lateral superior no lado da fissura. A substituição do incisivo lateral por um canino foi concluída após o enxerto ósseo alveolar secundário. Os modelos dentários foram obtidos entre dois e seis meses após a remoção do aparelho (idade média: 20,4 anos). Foram mensuradas nos dentes anteriores as variáveis: altura, largura, proporção e simetria da coroa, bem como as distâncias entre as bordas incisais, margens gengivais, angulação mesiodistal e inclinação vestibulolingual. O teste *t* pareado com correção *post-hoc* de Bonferroni foi usado para comparações entre os lados fissurados e não fissurados (*p*<0,05).

**Resultados:**

No lado fissurado, os caninos que substituíram os incisivos laterais ausentes apresentaram uma altura de coroa maior (0,77 mm) e uma largura aumentada (0,67 mm). Os primeiros pré-molares apresentaram uma altura de coroa menor (1,39 mm). Assimetrias foram observadas no nível gengival dos incisivos centrais e laterais, com maior coroa clínica no lado fissurado (0,61 e 0,81mm, respectivamente). Os incisivos centrais do lado fissurado estavam mais intruídos do que os contralaterais (2,12 mm).

**Conclusão:**

Os dentes anteriores superiores demonstraram diferenças de posição, tamanho e altura gengival entre os lados fissurado e não fissurado, após o fechamento do espaço da agenesia dos incisivos laterais superiores. Leves assimetrias na posição do dente e margem gengival nos dentes anteriores superiores devem ser esperadas após o tratamento ortodôntico em pacientes com fissura labiopalatina transforame unilateral.

## INTRODUCTION

Cleft lip and palate (CLP) is the most frequent craniofacial anomaly in humans.^1^ ^,^ ^2^ Individuals with CLP often show facial and dental esthetics impairment, resulting in low self-stem and difficulties in social interactions.^3^ Craniofacial rehabilitation aims to achieve adequate function and esthetics of the nose, lips and teeth, with the expectation to improve patient’s quality of life.^4^ ^,^ ^5^ Individuals with unilateral complete cleft lip and palate (UCLP) often have agenesis of the maxillary lateral incisors in the cleft area.^6^ ^,^ ^7^ The gold standard treatment plan is the mesial movement of maxillary canines after secondary alveolar bone graft (SABG) surgery in order to replace the missing lateral incisor.^8^ ^,^ ^9^

In non-cleft individuals, the orthodontic space closure of missing maxillary lateral incisors can provide excellent esthetics and functional results when multidisciplinary procedures are performed.^10^ ^-^ ^12^ The main advantages of space closure include avoiding the use of dental prosthesis and implants,^13^ ^,^ ^14^ and preventing long-term complications in gingival levels.^15^ ^-^ ^18^ Particularly in individuals with UCLP, there is evidence that orthodontic space closure contributes to the maintenance of the alveolar graft in the cleft area,^19^ ^,^ ^20^ providing improved esthetic outcomes, when compared to cases treated with dental implants or prosthetics in the missing lateral incisor area.^21^

Few studies have been conducted in order to assess the anterior dental esthetics of individuals with UCLP.^21^ ^-^ ^23^ Esper et al.^22^ reported that 13.3% of patients with UCLP considered their smile as esthetically unpleasant after complete dental rehabilitation. The most common reasons for the dissatisfaction included tooth shape, tooth positioning, tooth contour/color, lip shape and level.^22^ Another study in UCLP patients investigated the influence of various dental and surgical treatment options on gingival esthetics and oral health-related quality of life (OHRQoL). The authors concluded that natural teeth integrated into the cleft area showed more adequate esthetics and better quality of life perception.^21^

No previous study evaluated the degree of symmetry of maxillary anterior teeth in patients with UCLP after orthodontic treatment with space closure of absent lateral incisors. Thus, the aim of this study was to compare dental position and gingival parameters of maxillary anterior teeth in UCLP patients after orthodontic treatment with canine substitution on the cleft side lateral incisor agenesis. The null hypothesis was that cleft and noncleft sides would demonstrate similar positional and gingival features of anterior teeth after orthodontic treatment. 

## MATERIAL AND METHODS

This split-mouth study was approved by the Institutional Review Board of the Hospital for Rehabilitation of Craniofacial Anomalies, University of São Paulo (Protocol: 53829416.7.00005441). 

The sample size was calculate considering a capability to detect a 0.5-mm difference in the crown height of maxillary central incisors, with a standard deviation of 1.3mm, obtained from a pilot study, considering an alpha of 5% and a power of 80%. The minimal required sample size was 56 subjects.

Patients with UCLP from a single center, that finished comprehensive orthodontic treatment between 2011 and 2016, were screened. The inclusion criteria were: presence of final dental models (2 to 6 months after debonding); age varying from 15 to 30 years at debonding; lip repair performed between 3 and 6 months of age; palate repair performed between 12 and 18 months of age; secondary alveolar bone graft procedure performed with autogenous bone from the iliac crest between 9 and 12 years of age; presence of both maxillary lateral incisor and canine in the noncleft side (NCS); agenesis of the lateral incisor at the cleft side (CS); history of comprehensive orthodontic treatment including mesial movement of the maxillary canines and first premolars toward the alveolar grafted cleft site. The exclusion criteria were the presence of associated craniofacial syndromes; tooth loss in the maxillary arch; prosthetic rehabilitation in any of the maxillary anterior region teeth; and anterior teeth crown fracture. 

The final sample comprised post-debonding dental models of 57 patients (31 males and 26 females) with a mean age of 20.4 years. CS group was composed by the maxillary anterior teeth at the cleft side. NCS group comprised the maxillary anterior teeth at the non-cleft side. Pre-adjusted brackets (Capelozza prescription) were bonded in the center of clinical crown height, except the for cleft side canines, in which brackets were bonded slightly displaced toward cervical. The cleft side canine received their bracket with occlusocervical inverted position. Archwire bends were usually necessary during the finishing phase, and were performed when necessary. No reshaping of canine and premolars was performed until the end of the study. The average time of comprehensive orthodontic treatment was 4 years.

**Figure 1:**
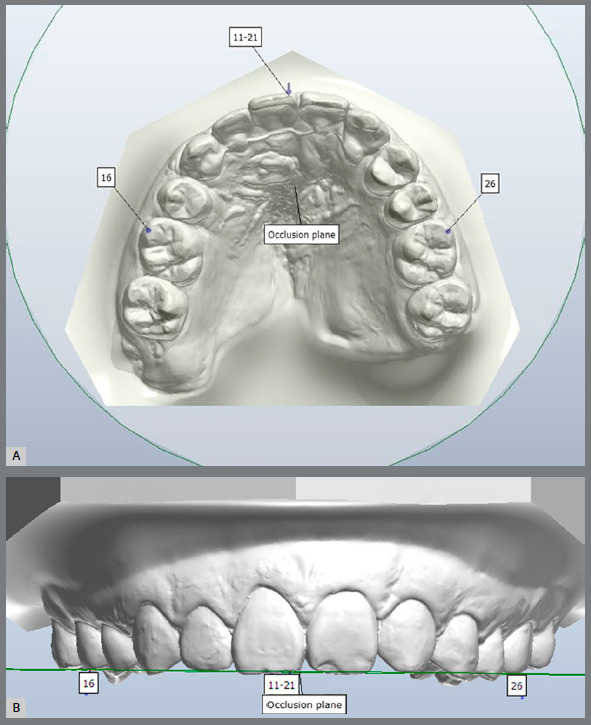
Occlusal plane (**OP**). A) Occlusal plane was defined as a plane passing bilaterally through the mesiobuccal cusp tip of the maxillary first molars and the mesioincisal point of the noncleft central incisor. B) Occlusal plane positioned parallel to the horizontal plane.

The posttreatment maxillary dental models of all subjects were scanned using a laser scanner 3Shape R700 3D (3Shape A/S, Copenhagen, Denmark). The images were saved in STL format and measured using the software OrthoAnalyzer 3D (3Shape A/S, Copenhagen, Denmark). An occlusal plane passing through the mesiobuccal cusp tip of the maxillary first molars and to the mesio-incisal point of the noncleft central incisor was positioned parallel to the horizontal plane in the model frontal view (Fig 1).

**Figure 2:**
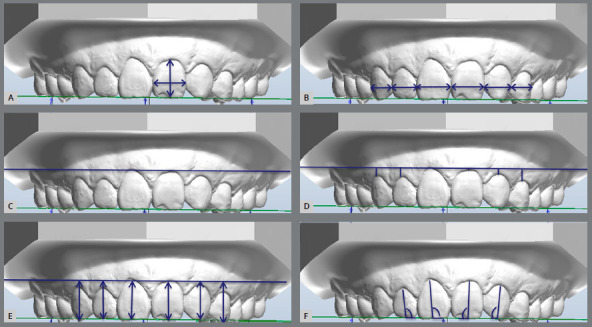
Digital dental model measurements using specific tools provided by the software. **A**) crown width (maximum distance between the mesial and distal contact points of the tooth) and height (distance between gingival zenith to the incisal edge). **B**) anterior view width (virtual width of the anterior teeth). **C**) A line (L) parallel to the occlusal plane (**OP**) and tangent to the gingival zenith of the U1 of the NCS in the UCLP was drawn to evaluate the gingival margin and incisal edges. **D**) Gingival margin (distance from the zenith of each tooth to L). **E**) Incisal level (distance from L to OP). **F**) Angulation of U1 and U2 (angle between the long axis and the OP).

**Figure 3:**
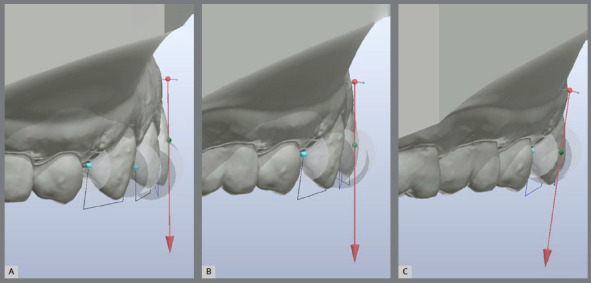
Crown labiolingual inclination of: A) Central incisor. B) lateral incisor and C) canine.

The following variables were measured in the anterior maxillary teeth: (*a*) crown height, (*b*) crown width, (*c*) crown width-to-height proportion, *(d*) mesiodistal dimension of anterosuperior teeth in a frontal perspective, (*e*) incisal edge symmetry between homologous teeth, (*f*) central-to-lateral incisal step, and central-to-canine incisal step, (*g*) gingival margin symmetry between homologous teeth, (*h*) central-to-lateral and central-to-canine gingival step, (*i*) crown angulation and (*x*) crown inclination (Figs 2 and 3). In the cleft side, canines were considered as lateral incisors, and first premolars were considered as canines. For measuring dental crown width and height, dental models were laterally rotated, in order to observe each tooth in a frontal perspective (Fig 2). 

For the other measurements, the models were fixed in the anterior frontal perspective, except for the crown inclination, which was measured from a distal view of each tooth crown.^24^ The width dimension of the crown was considered the greatest distance between the mesial and distal contact points of each tooth.^25^ The crown height was measured from the gingival zenith to the incisal edge.^25^ The ratio between width and height was calculated. 

Teeth were measured twice by one investigator, with a minimal interval of three weeks. Intra-rater agreement was assessed using intraclass correlation coefficients (ICC). 

Statistical analysis was performed considering the mean of the two measurements. The comparisons between the cleft and noncleft sides were evaluated using paired *t*-tests. Bonferroni correction for multiple comparisons was performed. The statistical analyses were conducted with statistical software Statistical Package for the Social Sciences v. 21 (SPSS Inc, Chicago, EUA). Clinical relevance was considered when statistical differences were greater than 0.5mm or 1 degree.

## RESULTS

Measurement agreements were considered good for linear variables (ICC≥0.75) and moderate for angular variables (0.4≤ICC<0,75). Mean differences between the linear and angular measurements were smaller than 0.5 mm and 1^o^, respectively.

The cleft side central incisors (U1) demonstrated a slightly greater mesiodistal width than contralaterals. Cleft side lateral incisors (U2, canines replacing missing laterals) showed a greater crown height (+0.77mm) and a greater mesiodistal width (+0.67mm), compared to non-cleft side (Table 1). Conversely, first premolars (U3) replacing the canines at the cleft side showed a smaller tooth crown height (-1.39mm) in comparison to non-cleft side canines, influencing the crown width/height ratio (Table 1). Cleft side first premolars had slightly smaller mesiodistal width (-0.35mm).

**Table 1:** Cleft and noncleft sides comparisons (paired *t*-tests).

Variable	Teeth	Cleft Side (n=57)		Non Cleft Side (n=57)		Diff.	p
		Mean	SD	Mean	SD		
Real width (mm)	U1	8.69	0.70	8.49	0.53	0.19	0.005*
	U2	7.75	0.50	7.07	0.59	0.67	<0.001*
	U3	7.32	0.56	7.68	0.52	-0.35	<0.001*
Height (mm)	U1	10.61	0.88	10.21	0.83	0.40	<0.001*
	U2	9.28	1.09	8.50	0.91	0.77	<0.001*
	U3	8.02	1.06	9.41	1.04	-1.39	<0.001*
Width/Height ratio	U1	0.82	0.09	0.83	0.08	-0.01	0.023
	U2	0.84	0.09	0.84	0.10	-0.00	0.079
	U3	0.92	0.13	0.82	0.09	0.10	<0.001*
Anterior view width (mm)	U1	8.45	0.89	8.41	0.49	0.04	0.712
	U2	6.36	0.26	6.07	0.49	0.28	<0.001*
	U3	5.03	0.55	5.36	0.62	-0.32	0.005*
Width ratio (mm)	U2/U1	0.76	0.11	0.72	0.06	0.03	0.019*
	U3/U2	0.79	0.09	0.89	0.13	-0.09	<0.001*
Gingival symmetry (mm)**	U1	0.62	0.83	0.00	0.00	0.62	<0.001*
	U2	-0.40	1.19	-1.21	1.19	0.81	<0.001*
	U3	-0.80	2.45	-1.34	0.94	0.54	0.156
Incisal symmetry (mm)***	U1	-0.16	0.44	0.00	0.00	-0.16	0.007*
	U2	-0.40	0.54	-0.33	0.48	-0.06	0.509
	U3	0.01	0.48	0.39	0.65	-0.37	0.002*
Gingival step (mm)**	U1 to U2	-1.02	1.16	-1.21	1.19	0.18	0.349
	U1 to U3	-1.42	2.83	-1.34	0.94	-0.07	0.854
Incisal step (mm)***	U1 to U2	-0.24	0.65	-0.33	0.48	0.09	0.303
	U1 to U3	0.18	0.54	0.39	0.65	-0.20	0.046
Angulation (degrees)	U1	0.70	4.18	2.82	3.57	-2.12	0.005*
	U2	2.81	4.28	4.32	3.96	-1.51	0.033
Inclination (degrees)****	U1	8.28	4.68	8.63	4.45	-0.34	0.388
	U2	7.41	4.22	8.36	4.65	-0.94	0.186
	U3	-5.59	3.02	-5.41	2.97	0.18	0.691

U1= Central Incisor; U2= Lateral Incisor; U3= Canine. *Statistically significant. ** Negative values indicate an occlusal displacement in relation to the reference line. *** Negative values indicate an apical position of the variable. **** Negative values indicate lingual inclination of the variable. After Bonferroni correction the level of significance considered was 1.66% for all measurements, except for width ratio, gingival step, incisal step and angulation, for which it was 2.5%.

A clinically significant asymmetry was observed for the gingival levels, which were more apically displaced in the cleft side for the central (+0.62mm) and lateral incisors (+0.81mm) (Table 1). A slight asymmetry was also observed for the incisal edge level of central incisor and canines, which were less extruded at the cleft side, without clinical relevance (Table 1). 

The non-cleft side central incisors were more mesio-angulated, compared to cleft side central incisors (+2.12º) (Table 1).

## DISCUSSION

This is the first study analyzing the magnitude of asymmetries between cleft and noncleft side after comprehensive orthodontic treatment in patients with complete unilateral cleft lip and palate. The cleft side has limitations for orthodontic finishing including the frequent prevalence of missing lateral incisors, the alveolar bone defect and the scars and fibrosis of the reconstructive plastic surgeries. The method of measuring digital dental models showed an adequate reproducibility. The angular measurements showed slightly less agreement than linear measurements, and these results are in accordance to previous studies.^26^ ^,^ ^27^ Digital dental models were previously validated to quantitative measurements.^28^ ^-^ ^31^

**Figure 4:**
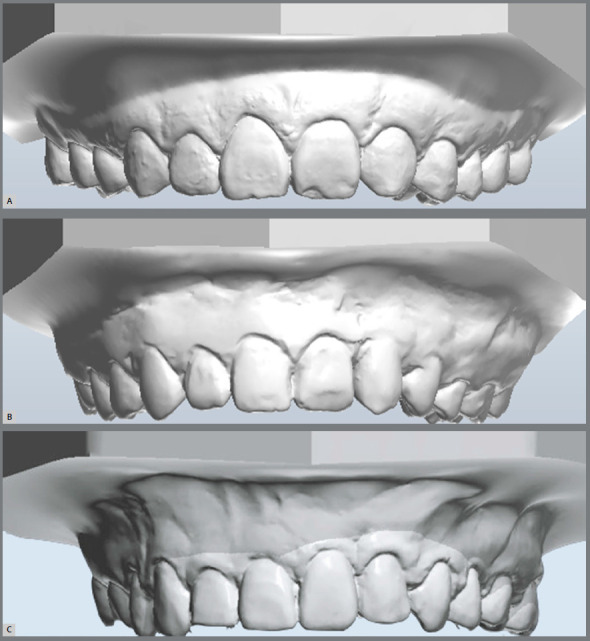
Three different subjects from the sample, with left complete unilateral cleft lip and palate, at the end of comprehensive orthodontic treatment. Observe dental and gingival asymmetries between cleft and noncleft sides.

An increased width for canines replacing lateral incisors on the cleft side was found, compared to the non-cleft side lateral incisor (Fig 4). A difficulty in achieving an acceptable esthetic outcome when replacing the lateral incisor by the canine is expected, due to differences in tooth mesiodistal sizes.^32^ Canine width on the cleft side can be reduced with interproximal enamel reduction to improve final esthetic results.^10^ ^-^ ^12^ ^,^ ^32^ However, there is a limit for interproximal reduction, to avoid dentin exposure. A previous study demonstrated that narrow canines were preferred in the position of lateral incisors.^33^ No mesiodistal tooth size asymmetries were found for the maxillary central incisors in this study, corroborating the study by Santos et al.^34^ However, other previous studies have reported smaller anterior tooth size on the cleft side in UCLP, compared to noncleft side.^35^ ^-^ ^37^ Tooth size and shape differences of the cleft side central incisors may be associated with the dental anomalies pattern.^38^ Clinician should observe each case individually, and an augmentation of the cleft-side central incisor can be recommended in case of clinically relevant asymmetries. Non-cleft patients with agenesis of lateral incisor also displayed a size reduction of both maxillary central incisors.^39^

In cases of missing lateral incisors followed by canine substitution in patients without oral clefts, premolar intrusion and canine extrusion can produce adequate gingival margin.^10^ ^-^ ^15^ Previous studies in noncleft patients recommended the protocol of extrusion of maxillary canines and intrusion of the first premolars for space closure of lateral incisor agenesis for remodeling of the gingival margin, achieving an adequate esthetical outcome.^11^ ^,^ ^15^ ^,^ ^40^ ^,^ ^41^ Although first premolars were slightly intruded and the canines extruded at the cleft side for improving the gingival margin, clinically relevant asymmetries between cleft and non-cleft side were still present after the orthodontic treatment. Crown heights were greater for U2 and smaller for U3 on the cleft side, in comparison to noncleft side (Fig 4). These differences were also reflected on the width/height ratio, gingival contour, gingival step and incisal step. The presence of asymmetries in dental and/or gingival margin might negatively influence the smile esthetics in patients with a high smile line.^42^ ^,^ ^43^ Additionally, incisal reduction of canines and augmentation of the first premolars was previously recommended.^12^

In the present study, an asymmetrical gingival level between cleft and non-cleft sides corroborated previous studies.^22^ ^,^ ^23^ ^,^ ^44^ At the cleft side, the central incisor showed a more apical displaced gingival margin (Figs 4B and 4C). The central incisor on the cleft side is usually severely rotate in UCLP before treatment. Orthodontic rotation of central incisors might produce buccal bone dehiscence.^44^ ^-^ ^46^ Furthermore, the flaps performed during secondary bone graft surgery may precipitate gingival recession in areas with buccal bone dehiscence.^44^ ^-^ ^46^ Canine replacing the lateral incisor on the cleft side showed an apical displaced gingival margin of 0.8mm, compared to non-cleft side (Fig 4). Canines replacing lateral incisors on the cleft side should be extruded by bonding the bracket toward cervical or using step downs associated with incisal reduction in cases where the exposure during the smile is evident.

Central incisors at the cleft side were more mesiodistally upright, when compared to the contralateral teeth (Fig 4C). The possible explanation is that cleft side maxillary central incisor usually demonstrated a crown angulation toward the alveolar cleft. The distal angulation of maxillary central incisors is a natural protection for the root to avoid the alveolar bone defect. The distal-angulation of cleft side central incisors may not have been completely corrected during comprehensive orthodontic treatment considering the gap between bracket-slot and archwires.^9^ A clinical solution would be bonding the bracket with more mesial angulation in the central incisor on the cleft side. 

In summary, this study suggests that some points should be considered by the clinician when the lateral incisor space is closed in UCLP. At the cleft side, canine width should be reduced, while the first premolar and central incisor can be augmented. Extrusion of maxillary canines, intrusion of the first premolars and labial crown torque of the maxillary canines should be performed during mechanics. Additionally, the symmetry of the mesiodistal angulation of maxillary central incisors should be checked before debonding. A limitation of this study was the absence of tooth reshaping at the time of the evaluation. However, the orthodontic finishing was analyzed without the influence of other multiple factors that could significantly affect the final esthetic result. Future studies should assess the self-perception of the smile after orthodontic treatment in subjects with UCLP.

## CONCLUSIONS

» The null hypothesis was rejected. Maxillary anterior teeth demonstrated positional and gingival asymmetries between cleft and noncleft side in individuals with UCLP treated with closure of maxillary lateral incisor agenesis space.
» Canines replacing missing lateral incisors had a larger crown height and width, and first premolars showed a shorter crown height.
» Asymmetries were observed in the gingival level of central and lateral incisors, with a greater clinical crown at the cleft side.
» Cleft side central incisors were more upright than contralaterals.

## References

[1] Ranta R (1986). A review of tooth formation in children with cleft
lip/palate. Am J Orthod Dentofacial Orthop.

[2] Cleft Palate Craniofac J (2004). Global strategies to reduce the health care burden of
craniofacial anomalies report of WHO meetings on international collaborative
research on craniofacial anomalies. Cleft Palate Craniofac J.

[3] Meyer-Marcotty P, Stellzig-Eisenhauer A (2009). Dentofacial self-perception and social perception of adults with
unilateral cleft lip and palate. J Orofac Orthop.

[4] Munz SM, Edwards SP, Inglehart MR (2011). Oral health-related quality of life, and satisfaction with
treatment and treatment outcomes of adolescents/young adults with cleft
lip/palate an exploration. Int J Oral Maxillofac Surg.

[5] Marcusson A, Akerlind I, Paulin G (2001). Quality of life in adults with repaired complete cleft lip and
palate. Cleft Palate Craniofac J.

[6] Halpern RM, Noble J (2010). Location and presence of permanent teeth in a complete bilateral
cleft lip and palate population. Angle Orthod.

[7] Lourenço Ribeiro L, Teixeira Das Neves L, Costa B, Ribeiro Gomide M (2003). Dental anomalies of the permanent lateral incisors and prevalence
of hypodontia outside the cleft area in complete unilateral cleft lip and
palate. Cleft Palate Craniofac J.

[8] Cassolato SF, Ross B, Daskalogiannakis J, Noble J, Tompson B, Paedo D (2009). Treatment of dental anomalies in children with complete
unilateral cleft lip and palate at SickKids hospital,
Toronto. Cleft Palate Craniofac J.

[9] Freitas JA, Garib DG, Oliveira M, Lauris RCMC, Almeida ALPF, Neves LT (2012). Rehabilitative treatment of cleft lip and palate experience of
the Hospital for Rehabilitation of Craniofacial Anomalies-USP (HRAC-USP) -
part 2: pediatric dentistry and orthodontics. J Appl Oral Sci.

[10] Rosa M, Zachrisson BU (2001). Integrating esthetic dentistry and space closure in patients with
missing maxillary lateral incisors. J Clin Orthod.

[11] Rosa M, Zachrisson BU (2007). Integrating space closure and esthetic dentistry in patients with
missing maxillary lateral incisors. J Clin Orthod.

[12] Zachrisson BU, Rosa M, Toreskog S (2011). Congenitally missing maxillary lateral incisors: canine
substitution. Point. Am J Orthod Dentofacial Orthop.

[13] Tuverson DL (1970). Orthodontic treatment using canines in place of missing maxillary
lateral incisors. Am J Orthod.

[14] Thordarson A, Zachrisson BU, Mjör IA (1991). Remodeling of canines to the shape of lateral incisors by
grinding a long-term clinical and radiographic evaluation. Am J Orthod Dentofacial Orthop.

[15] Rosa M, Zachrisson BU (2010). The space-closure alternative for missing maxillary lateral
incisors: an update. J Clin Orthod.

[16] Thilander B, Odman J, Lekholm U (2001). Orthodontic aspects of the use of oral implants in adolescents a
10-year follow-up study. Eur J Orthod.

[17] Bernard JP, Schatz JP, Christou P, Belser U, Kiliaridis S (2004). Long-term vertical changes of the anterior maxillary teeth
adjacent to single implants in young and mature adults A retrospective
study. J Clin Periodontol.

[18] Dueled E, Gotfredsen K, Trab Damsgaard M, Hede B (2009). Professional and patient-based evaluation of oral rehabilitation
in patients with tooth agenesis. Clin Oral Implants Res.

[19] Liao YF, Huang CS (2015). Presurgical and postsurgical orthodontics are associated with
superior secondary alveolar bone grafting outcomes. J Craniomaxillofac Surg.

[20] Schultze-Mosgau S, Nkenke E, Schlegel AK, Hirschfelder U, Wiltfang J (2003). Analysis of bone resorption after secondary alveolar cleft bone
grafts before and after canine eruption in connection with orthodontic gap
closure or prosthodontic treatment. J Oral Maxillofac Surg.

[21] Stelzle F, Rohde M, Oetter N, Krug K, Riemann M, Adler W (2017). Gingival esthetics and oral health-related quality of life in
patients with cleft lip and palate. Int J Oral Maxillofac Surg.

[22] Esper LA, Sbrana MC, Ribeiro IW, de Siqueira EN, de Almeida AL (2009). Esthetic analysis of gingival components of smile and degree of
satisfaction in individuals with cleft lip and palate. Cleft Palate Craniofac J.

[23] Esper LA, Sbrana MC, Cunha MJ, Moreira GS, de Almeida AL (2012). Esthetic composition of smile in individuals with cleft lip,
alveolus, and palate visibility of the periodontium and the esthetics of
smile. Plast Surg Int.

[24] Andrews LF (1972). The six keys to normal occlusion. Am J Orthod.

[25] Sah SK, Zhang HD, Chang T, Dhungana M, Acharya L, Chen LL (2014). Maxillary anterior teeth dimensions and proportions in a central
mainland chinese population. Chin J Dent Res.

[26] Massaro C, Losada C, Cevidanes L, Yatabe M, Garib D, Lauris JRP (2020). Comparison of linear and angular changes assessed in digital
dental models and cone-beam computed tomography. Orthod Craniofac Res.

[27] Dastoori M, Bouserhal JP, Halazonetis DJ, Athanasiou AE (2018). Anterior teeth root inclination prediction derived from digital
models A comparative study of plaster study casts and CBCT
images. J Clin Exp Dent.

[28] Al-Mashraqi AA, Alhammadi MS, Gadi AA (2021). Accuracy and reproducibility of permanent dentitions and dental
arch measurements comparing three different digital models with a plaster
study cast. Int J Comput Dent.

[29] Komuro A, Yamada Y, Uesugi S, Terashima H, Kimura M, Kishimoto H (2021). Accuracy and dimensional reproducibility by model scanning,
intraoral scanning, and CBCT imaging for digital implant
dentistry. Int J Implant Dent.

[30] Ferreira JB, Christovam IO, Alencar DS, Motta AFJ, Mattos CT, Cury-Saramago A (2017). Accuracy and reproducibility of dental measurements on
tomographic digital models a systematic review and
meta-analysis. Dentomaxillofac Radiol.

[31] Sousa MV, Vasconcelos EC, Janson G, Garib D, Pinzan A (2012). Accuracy and reproducibility of 3-dimensional digital model
measurements. Am J Orthod Dentofacial Orthop.

[32] Kokich VO, Kinzer GA (2005). Managing congenitally missing lateral incisors Part I: Canine
substitution. J Esthet Restor Dent.

[33] Brough E, Donaldson AN, Naini FB (2010). Canine substitution for missing maxillary lateral incisors: the
influence of canine morphology, size, and shade on perceptions of smile
attractiveness. Am J Orthod Dentofacial Orthop.

[34] dos Santos PB, Garib DG, Janson G, Assis VH (2015). Association between tooth size and interarch relationships in
children with operated complete unilateral cleft lip and
palate. Prog Orthod.

[35] Dindaroglu F, Duran GS, Dogan S (2019). Dental crown symmetry in unilateral cleft lip and palate patients
A three-dimensional analysis on digital dental models. Orthod Craniofac Res.

[36] Lewis BR, Stern MR, Willmot DR (2008). Maxillary anterior tooth size and arch dimensions in unilateral
cleft lip and palate. Cleft Palate Craniofac J.

[37] Antonarakis GS, Tsiouli K, Christou P (2013). Mesiodistal tooth size in non-syndromic unilateral cleft lip and
palate patients a meta-analysis. Clin Oral Investig.

[38] Germec Cakan D, Nur Yilmaz RB, Bulut FN, Aksoy A (2018). Dental anomalies in different types of cleft lip and palate is
there any relation?. J Craniofac Surg.

[39] Mirabella AD, Kokich VG, Rosa M (2012). Analysis of crown widths in subjects with congenitally missing
maxillary lateral incisors. Eur J Orthod.

[40] Rosa M, Lucchi P, Ferrari S, Zachrisson BU, Caprioglio A (2016). Congenitally missing maxillary lateral incisors Long-term
periodontal and functional evaluation after orthodontic space closure with
first premolar intrusion and canine extrusion. Am J Orthod Dentofacial Orthop.

[41] Rosa M, Zachrisson B, Nanda R (2015). Esthetics and Biomechanics in Orthodontics.

[42] Kokich VO, Kokich VG, Kiyak HA (2006). Perceptions of dental professionals and laypersons to altered
dental esthetics asymmetric and symmetric situations. Am J Orthod Dentofacial Orthop.

[43] Kokich VO, Kiyak HA, Shapiro PA (1999). Comparing the perception of dentists and lay people to altered
dental esthetics. J Esthet Dent.

[44] Zhu S, Chen Z (2013). Association between gingival recession and proclination of
maxillary central incisors near the cleft in patients with unilateral cleft
lip and palate A retrospective case-control study. Am J Orthod Dentofacial Orthop.

[45] Fuhrmann RA, Bücker A, Diedrich PR (1995). Assessment of alveolar bone loss with high resolution computed
tomography. J Periodontal Res.

[46] Amodeo G, Scopelliti D (2018). Mucosal dehiscence after alveolar bone graft in
cleft. J Craniofac Surg.

